# Function of miR-146a-5p in Tumor Cells As a Regulatory Switch between Cell Death and Angiogenesis: Macrophage Therapy Revisited

**DOI:** 10.3389/fimmu.2017.01931

**Published:** 2018-01-05

**Authors:** Elina Simanovich, Vera Brod, Maya M. Rahat, Michal A. Rahat

**Affiliations:** ^1^Immunotherapy Laboratory, Carmel Medical Center, Haifa, Israel; ^2^The Ruth and Bruce Rappaport Faculty of Medicine, Technion-Israel Institute of Technology, Haifa, Israel

**Keywords:** miR-146a, antagomir, nitric oxide, EMMPRIN/CD147, tumor angiogenesis, tumor cell death, macrophage therapy, adoptive transfer

## Abstract

Tumors survive and progress by evading killing mechanisms of the immune system, and by generating a tumor microenvironment (TME) that reprograms macrophages *in situ* to produce factors that support tumor growth, angiogenesis, and metastasis. We have previously shown that by blocking the translation of the enzyme inducible nitric oxide synthase (iNOS), miR-146a-5p inhibits nitric oxide (NO) production in a mouse renal carcinoma cell line (RENCA), thereby endowing RENCA cells with resistance to macrophage-induced cell death. Here, we expand these findings to the mouse colon carcinoma CT26 cell line and demonstrate that neutralizing miR-146a-5p’s activity by transfecting both RENCA and CT26 cells with its antagomir restored iNOS expression and NO production and enhanced susceptibility to macrophage-induced cell death (by 48 and 25%, respectively, *p* < 0.001). Moreover, miR-146a-5p suppression simultaneously inhibited the expression of the pro-angiogenic protein EMMPRIN (threefolds, *p* < 0.001), leading to reduced MMP-9 and vascular endothelial growth factor secretion (twofolds and threefolds, respectively, *p* < 0.05), and reduced angiogenesis, as estimated by *in vitro* tube formation and scratch assays. When we injected tumors with pro-inflammatory-stimulated RAW 264.7 macrophages together with i.v. injection of the miR-146a-5p antagomir, we found inhibited tumor growth (sixfolds, *p* < 0.001) and angiogenesis (twofolds, *p* < 0.01), and increased apoptosis (twofolds, *p* < 0.01). This combination therapy increased nitrites and reduced TGFβ concentrations in tumor lysates, alleviated immune suppression, and allowed enhanced infiltration of cytotoxic CD8^+^ T cells. Thus, miR-146a-5p functions as a control switch between angiogenesis and cell death, and its neutralization can manipulate the crosstalk between tumor cells and macrophages and profoundly change the TME. This strategy can be therapeutically utilized in combination with the macrophage therapy approach to induce the immune system to successfully attack the tumor, and should be further explored as a new therapy for the treatment of cancer.

## Introduction

By secreting a myriad of chemoattractants and growth factors, tumor cells actively recruit macrophages into the tumor mass and reprogram them *in situ* to produce elevated levels of growth factors, pro-angiogenic factors, and anti-inflammatory cytokines that collectively promote tumor growth and metastasis and mediate evasion of immune recognition (1–4).

One of the hallmarks of pro-inflammatory macrophages or M1-activated macrophages is the high expression of the enzyme inducible nitric oxide synthase (iNOS) that generates high amounts of the cytotoxic molecule nitric oxide (NO), as well as other cytotoxic molecules (e.g., TNFα) that serve as a killing mechanism (5). However, the infiltrating macrophages that encounter the tumor microenvironment (TME) lose this capability as they are rapidly skewed toward an activation mode approximating the M2-activation mode (6).

The role of NO production in the TME is very complex and depends on the relative concentrations generated by both macrophages and tumor cells. Tumor-associated macrophages and myeloid-derived suppressor cells, both of which are M2-like activated, secrete low levels of NO that are pro-angiogenic and immunosuppressive (7, 8). Tumor cells can also produce low amounts of NO (9), however, it has been demonstrated that in some types of tumors, tumor cells of higher grade and stage as well as metastatic cells tend to reduce or completely lose their iNOS expression in order to resist immune killing (10). We have recently demonstrated that in the mouse renal cell carcinoma cell line RENCA, a specific microRNA molecule—miR-146a-5p—mediates the translational inhibition of iNOS (11).

In many tumors, the expression of the potent pro-angiogenic factors vascular endothelial growth factor (VEGF) and matrix metalloproteinase-9 (MMP-9) is upregulated by the protein extracellular matrix metalloproteinase inducer (EMMPRIN/CD147). EMMPRIN is a surface multifunctional protein, expressed on both tumor and stroma cells (12, 13), that can induce the expression of both VEGF and MMP-9 and enhance angiogenesis, probably through homophilic interactions (14, 15). EMMPRIN is also found secreted, and its overexpression in many types of tumors was correlated to enhanced levels of VEGF and MMP-9 and to increased invasiveness (16, 17). We have recently demonstrated, in the human renal and breast tumor cells lines A498 and MCF7, that neutralization of miR-146a-5p reduces the expression of EMMPRIN in these cells (17).

The cytotoxic capacity of macrophages and their ability to home to sites of inflammation, including cancerous lesions, rendered these cells a favorable target for therapy. However, once recruited into the tumor, the immunosuppressive TME polarizes and activates those cells to promote tumor growth. One of the therapeutic strategies used was to activate autologous immune cells *ex vivo* with IFNγ or combination of LPS and IFNγ, and then reinfuse then back into the patient. Such clinical trials were well-tolerated and showed feasibility, safety, and minimal adverse effects of the treatment (18–20). However, they also demonstrated a limited anti-tumoral activity, suggesting that the activation was not sufficient to overcome the immunosuppressive TME (21). As part of the TME, the ability of hypoxia, which is a dominant characteristic of solid tumors, to shift M1-activated macrophages to M2-like activated macrophages, and in particular to inhibit iNOS activity, certainly contributes to this failure (6, 10, 11). Thus, the macrophage therapy approach has been abandoned, until a way was found to overcome the influence of the immunosuppressive TME.

MicroRNA are small non-coding RNA strands that regulate gene expression, and their aberrant expression play a crucial role in cancerous diseases. Therefore, several therapeutic approaches designed to regulate their expression were developed, including antisense oligonucleotides (antagomirs). The RNA backbone of these antagomirs is often chemically modified [by replacing the oxygen in the phosphate group with sulfur, adding 2′-*O*-methyl group to non-bridging oxygen, connecting the 2′-oxygen to the 4′-carbon to lock the bridge-locked nucleic acids (LNA), or by adding a peptide], to increase their stability, specificity, and binding affinity [reviewed in Ref. (22, 23)]. Such modifications enabled the systemic intravenous administration of antagomirs in cancer, cardiovascular, and other preclinical disease models (24–26), which resulted in a specific reduction in the expression of the tested miRNAs and a marked effect on the expression of their target genes. This opened the door for microRNA-based therapy approaches, where specific miRNAs can be suppressed as needed.

Since we separately demonstrated the ability of miR-146a-5p to regulate the expression of two of the key mediators of angiogenesis and death, EMMPRIN and iNOS, we now ask whether miR-146a-5p can serve as regulatory switch between apoptosis and angiogenesis through its simultaneous and opposite effects on iNOS and EMMPRIN expression in the tumor cell. More importantly, we explore the possible use of miR-146a-5p neutralization as a possible new therapeutic approach for the inhibition of tumor growth in combination with the adoptive transfer of stimulated macrophages.

## Results

### Pro-inflammatory Stimulation of RENCA and CT26 Cells Elevates the Expression of miR-146a-5p and the Transcription, but Not the Expression, of iNOS

**Figure 1:**
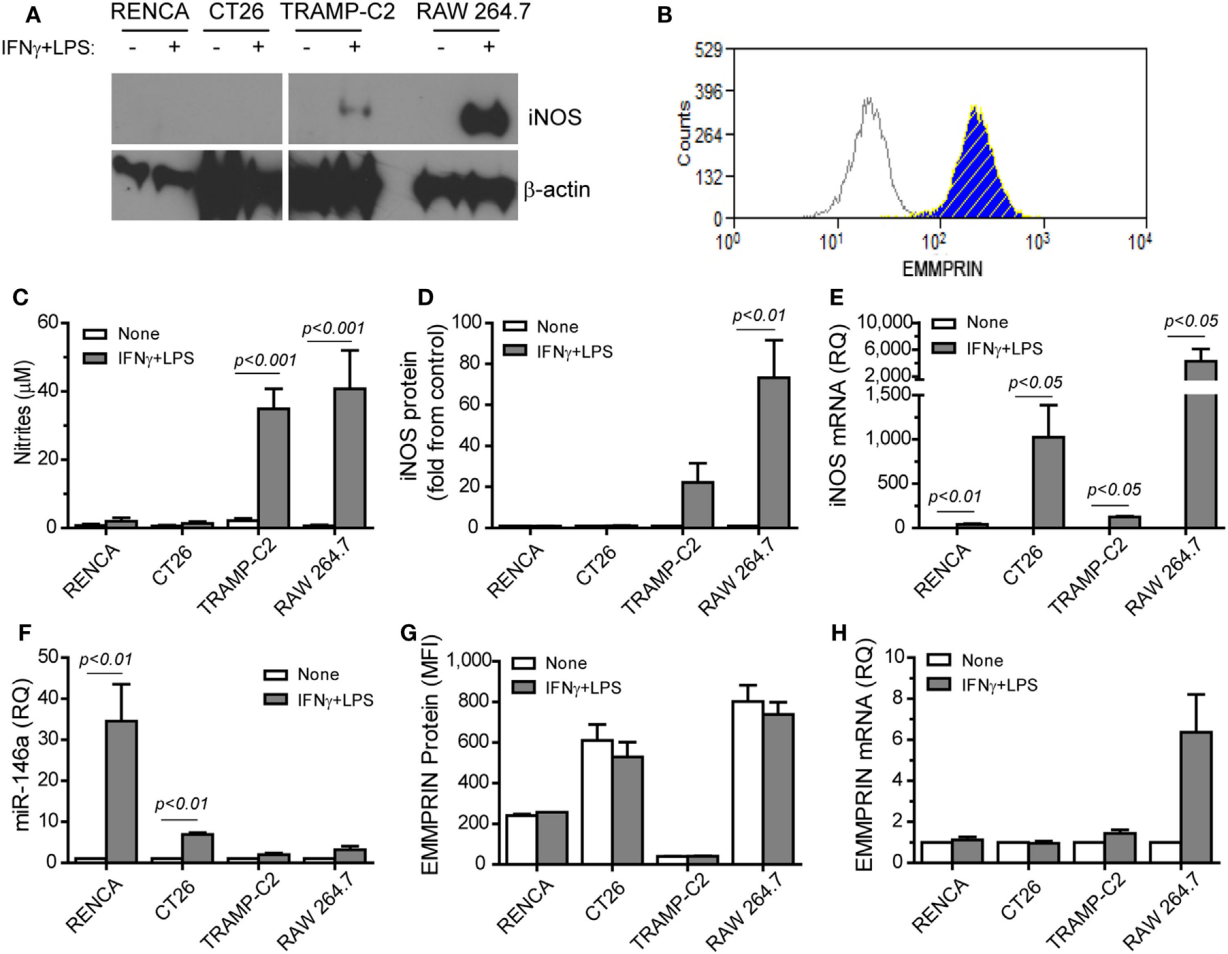
Translational inhibition of inducible nitric oxide synthase (iNOS) is reversely correlated with miR-146a expression. RENCA, CT26, and TRAMP-C2 tumor cells lines (10^6^ cells), and RAW 264.7 macrophage-like cells (10^6^ cells) were incubated with or without the combined stimulation of IFNγ (100 U/ml) and LPS (1 µg/ml) for 24 h. **(A)** A representative western blot analysis for iNOS expression and **(B)** a representative histogram depicting EMMPRIN expression in RENCA cells (light gray, isotype control; blue, no stimulation; hatched yellow, with the combined stimulation). **(C)** Accumulation of nitrites, the stable product of nitric oxide (NO), reflecting inducible nitric oxide synthase (iNOS) activity; **(D)** densitometric analysis of western blots for iNOS protein expression; **(E)** iNOS mRNA accumulation; **(F)** accumulation of miR-146a-5p expression; **(G)** mean fluorescence of EMMPRIN protein expression; **(H)** accumulation of EMMPRIN mRNA (*n* = 5–6 in each group).

The combination of IFNγ and LPS is the strongest known stimulation for mouse iNOS expression and NO production in many cell types, but not in all. Moreover, the effects of this combination on EMMPRIN expression have not been explored. We used the macrophage-like cell line RAW 264.7 as a positive control (Figure 1) and compared it to the three mouse tumor cell lines, the renal (RENCA), colon (CT26), and prostate (TRAMP-C2) carcinoma cell lines. We show here that the TRAMP-C2 cells responded to the combined stimulation by increasing their iNOS mRNA and protein expression (22-folds, *p* < 0.01), as well as their NO production (17-folds, *p* < 0.001). By contrast, the CT26 colon tumor cells did not express the protein or produced nitrites (Figures 1A,C,D), similar to the RENCA cells (11), despite elevated iNOS mRNA levels (Figure 1E).

Since iNOS mRNA was increased in all three cell types, but protein expression was not, we reasoned that a post-transcriptional regulation of iNOS exists in CT26 and RENCA cells, but not in TRAMP-C2 cells. Indeed, the combined stimulation increased the expression of miR-146a-5p only in the RENCA and CT26 cells (by 34- and 7-folds, *p* < 0.01, Figure 1F). We also observed that the combined stimulation did not change the accumulation of EMMPRIN mRNA or protein in the three tumor cell lines (Figures 1B,G,H). Thus, the expression of iNOS is inversely correlated with miR-146a-5p expression in the three tumor cells, and EMMPRIN expression does not correlate to the stimulation or to miR-146a-5p expression, probably as it is already maximally expressed.

### Neutralization of miR-146a-5p by Its Antagomir Restores iNOS Expression and Reduces EMMPRIN Expression

**Figure 2:**
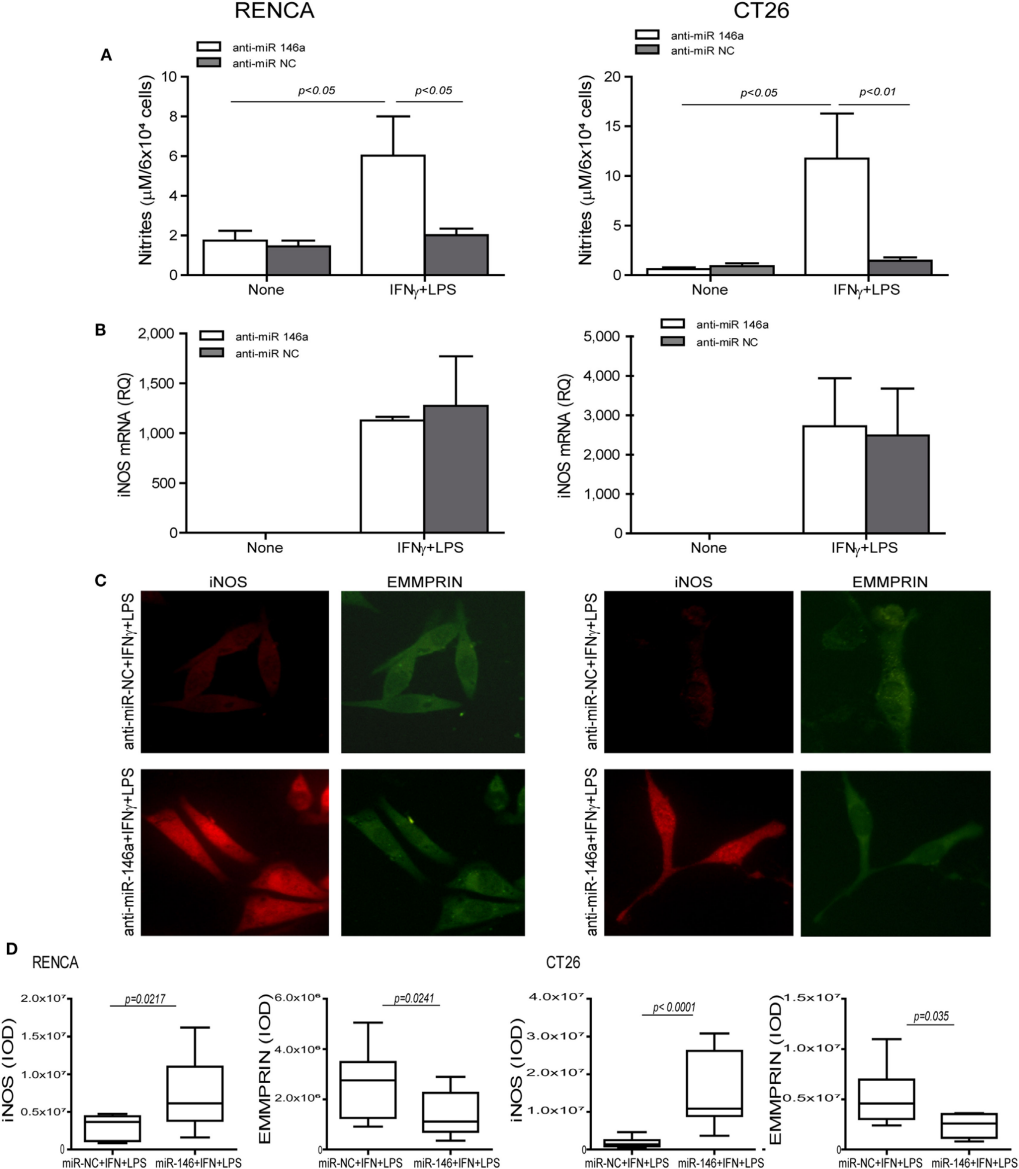
Translational inhibition of inducible nitric oxide synthase (iNOS) in RENCA and CT26 cells is mediated by miR-146a-5p. RENCA or CT26 cells (5 × 10^4^ cells) were transfected with anti-miR-146a-5p antagomir or with its negative control (anti-miR-NC) 24 h before they were stimulated with IFNγ (100 U/ml) and LPS (1 µg/ml) for 24 h. **(A)** Accumulation of nitrites and **(B)** accumulation of iNOS mRNA suggest a post-translational regulation. **(C)** Immunofluorescent staining **(D)** and their quantitation of cells transfected with the negative control (upper panel) or with the miR-146a-5p antagomir (lower panel) and stimulated with IFNγ and LPS, and were stained for iNOS (red staining) or EMMPRIN (green staining), magnification 200×. Only cells that were both transfected with anti-miR-146a-5p and incubated with the combined stimulation restored iNOS expression and accumulated nitrites, whereas the constitutive expression of EMMPRIN was reduced with the antagomir, regardless of the combined stimulation (*n* = 4 in all groups).

To demonstrate that miR-146a suppresses iNOS expression in CT26 tumor cells, we neutralized its activity by transfecting the cells with its antagomir, as we have done before in RENCA cells (11). We used the mirVana™ anti-miR-146a-5p inhibitor, a potent, chemically modified single-stranded RNA molecule with a sequence complementary to that of miR-146a-5p (anti-miR-146a-5p). The combined stimulation markedly elevated iNOS mRNA in both cell lines when transfected by either the antagomir or its negative control (*p* < 0.05, Figure 2B). However, the negative control did not induce iNOS protein expression or NO production in both cell lines, even in the presence of the combined stimulation, as evident by immunofluorescence (Figure 2C, red staining, and the relevant parts of Figure 2D) and nitrite accumulation (Figure 2A). Likewise, transfection with the antagomir in the absence of the combined stimulation did not induce iNOS expression (Figure 2A). Only transfection with the antagomir in the presence of the combined stimulation restored iNOS protein induction and NO production (threefolds and eightfolds for RENCA and CT26, respectively, *p* < 0.05, Figures 2A,C,D). Thus, iNOS expression and NO production in tumor cells require a strong pro-inflammatory stimulation, together with neutralization of miR-146a-5p activity, in both RENCA and CT26 cells.

**Figure 3:**
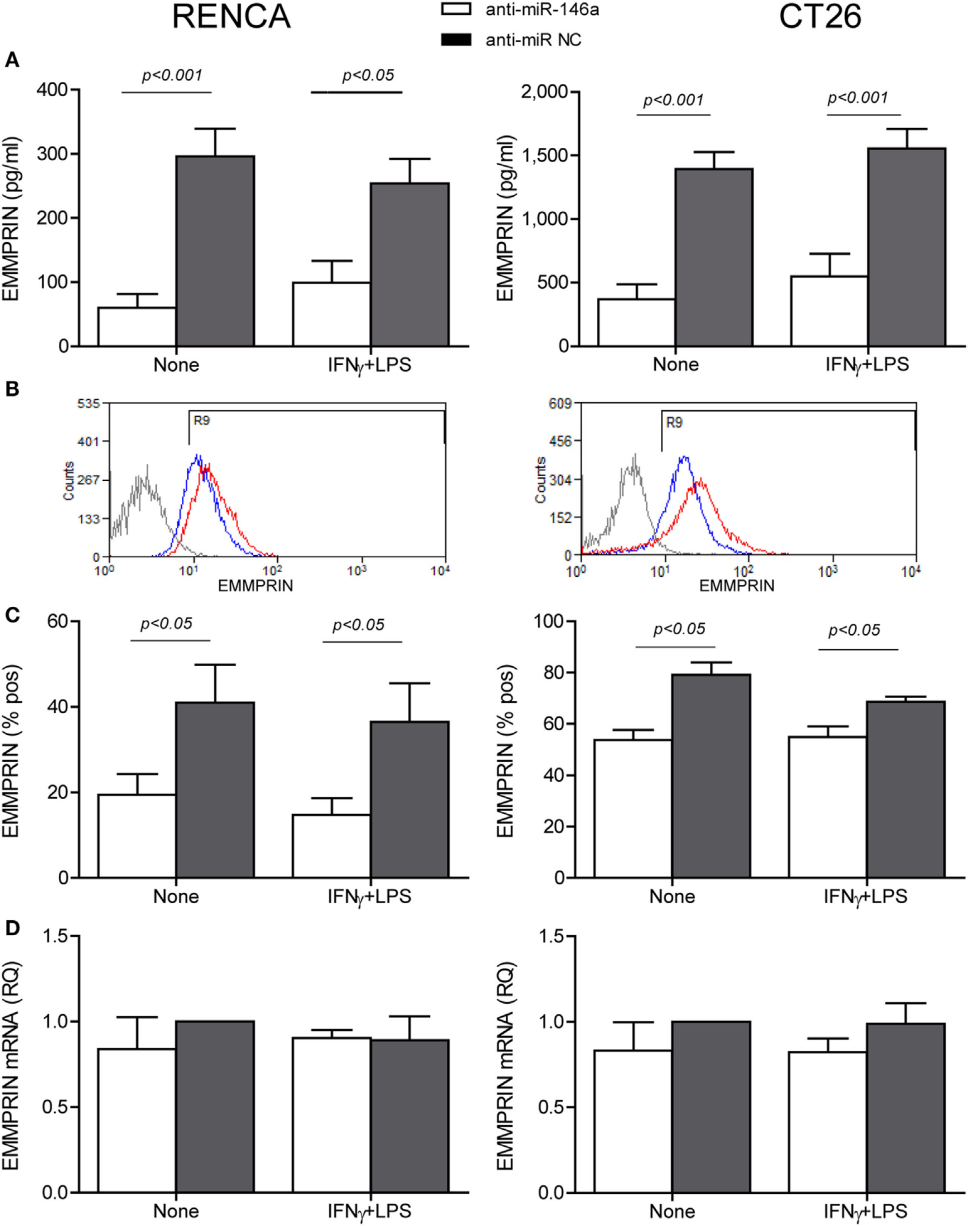
Anti-miR-146a inhibits EMMPRIN expression in RENCA and CT26 cells. RENCA or CT26 cells (5 × 10^4^ cells) were transfected and stimulated as described in Figure 2. **(A)** Accumulation of soluble EMMPRIN measured by ELISA; **(B)** representative histograms of surface EMMPRIN expression (gray line, isotype control; red line, cells transfected with the anti-miR-NC; blue line, cells transfected with anti-miR-146a-5p), and **(C)** percentage of positive cells expressing membranal EMMPRIN. **(D)** Accumulation of EMMPRIN mRNA detected by quantitative real-time PCR. The difference between the mRNA and protein expression levels suggests a post-translational regulation (*n* = 4–5 in each group).

To explore the effects of the combined stimulation and miR-146a-5p on EMMPRIN expression in the same transfected cells, we stained for EMMPRIN as well. EMMPRIN was constitutively expressed in both RENCA and CT26 cells, and no change was visible upon incubation with the combined stimulation. However, transfection of the antagomir resulted in a decrease in the intensity of EMMPRIN staining compared to the cells transfected with the negative control (Figure 2C, green staining, and the relevant parts of Figure 2D). This effect was also quantified by evaluating the amounts of the secreted protein (3-fold decrease for both cell lines, *p* < 0.05, Figure 3A), and by assessing the membranal expression of the protein by flow cytometry (1.5- to 2-fold decrease, *p* < 0.05, Figures 3B,C). However, EMMPRIN mRNA was unaffected by the combined stimulation or the transfection of the antagomir (Figure 3D). Thus, EMMPRIN expression is also post-transcriptionally regulated in both tumor cell lines.

### *In Vitro* Neutralization of miR-146a-5p by Its Antagomir Leads to Enhanced Tumor Cell Death and Reduced Angiogenesis

**Figure 4:**
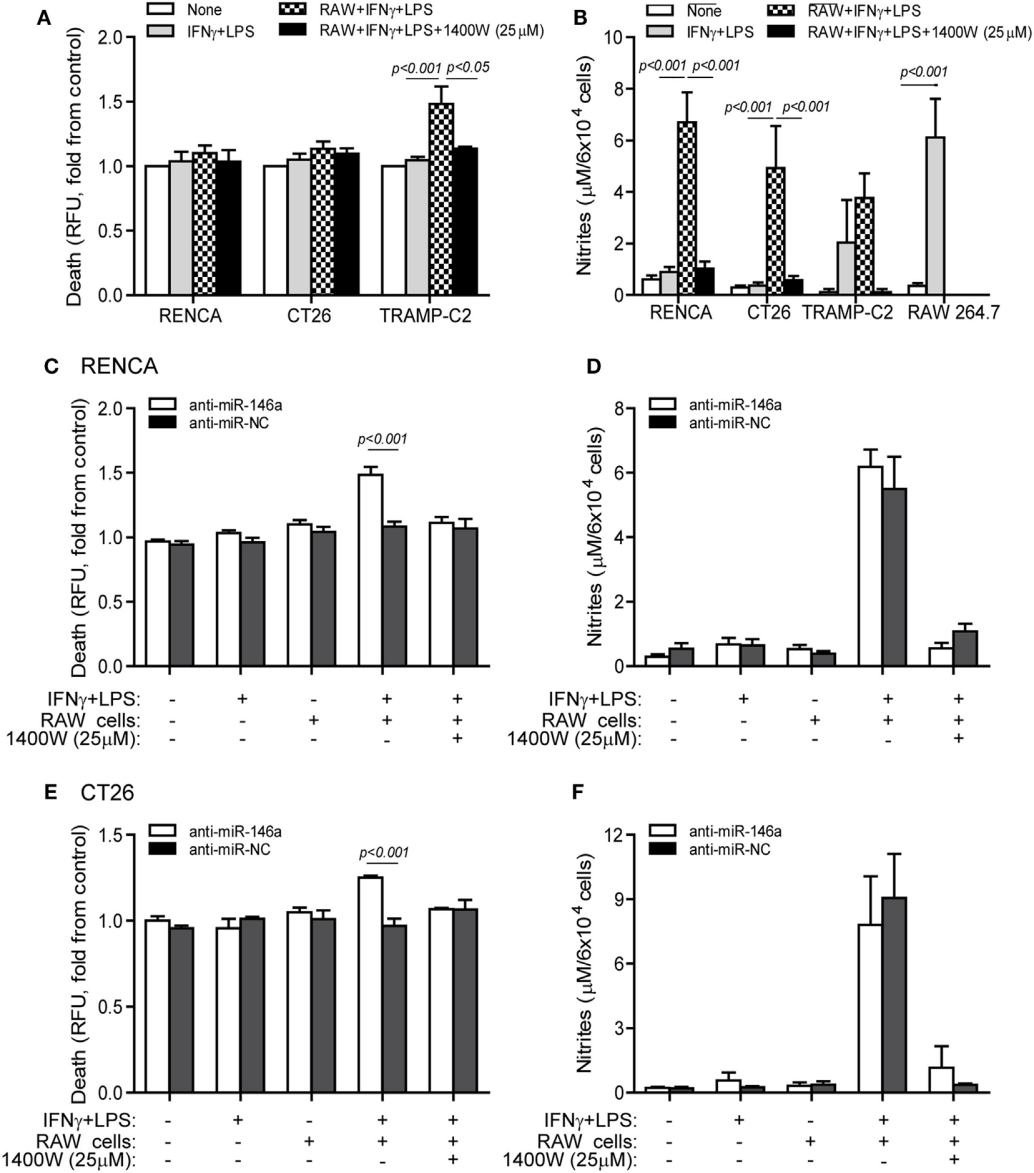
Cytotoxic activity of macrophages depends on endogenous nitric oxide (NO) production in the tumor cells. **(A,B)** RENCA, CT26, or TRAMP-C2 cells (5 × 10^4^ cells) were labeled with Cell Tracker Orange, and then cocultured for 24 h with unlabeled RAW 264.7 cells at a 2:1 ratio, with or without IFNγ (100 U/ml) and LPS (1 µg/ml), and with or without the addition of the 1400W inducible nitric oxide synthase (iNOS) inhibitor (25 µM). **(A)** Supernatants were collected and fluorescence was determined as a measure for tumor cell death, and calculated as fold change compared to non-stimulated cells. **(B)** Nitrite accumulation was measured in the supernatants. **(C–F)** RENCA and CT26 cells were labeled as before, and transfected with either anti-miR-146a-5p or its negative control (anti-miR-NC) 24 h before exposure to the combined stimulation and RAW 264.7 cells. Fluorescence was determined and reflected **(C)** RENCA cell death, and **(E)** CT26 cell death. Nitrite accumulation in the supernatants of **(D)** RENCA cells and **(F)** CT26 cells. TRAMP-C2 cells that produced endogenous NO upon stimulation exhibited increased death when cocultured with the macrophages, whereas RENCA and CT26 cells did not die despite the high NO accumulation, unless they were first transfected with anti-miR-146a-5p (*n* = 6 in all groups).

Some tumor cells lose iNOS expression in order to escape immune-mediated death (10), and we have shown that despite the high levels of NO secreted by stimulated macrophages, they cannot kill RENCA cells that do not express iNOS, unless iNOS expression is restored by transfecting the cells with the miR-146a antagomir (11). When RENCA, CT26, or TRAMP-C2 cells were cocultured with the RAW 264.7 macrophages in the presence of the combined stimulation, only TRAMP-C2 cells exhibited increased death [48 ± 13% increase (Figure 4A), *p* < 0.001], despite the high NO levels accumulated in all cocultures (Figure 4B). Cell death was abrogated by the addition of the selective iNOS inhibitor 1,400W (*p* < 0.05), suggesting that it was NO dependent.

To show that the induction of death by pro-inflammatory macrophages depends on the activity of miR-146a in the tumor cells, we next cocultured RAW 264.7 macrophages with RENCA (Figure 4C) or CT26 (Figure 4E) cells transfected with anti-miR-146a, and assessed tumor cell death relative to cells transfected with the negative control. RENCA cell death was increased by 48 ± 6% (*p* < 0.001) and CT26 cell death was increased 25 ± 1.3% (*p* < 0.001), only when anti-miR-146a-5p was introduced and when the combined stimulation was present. Again, this was abolished by the (1400W) iNOS inhibitor, demonstrating an NO-dependent effect. Despite the difference in cell death, we did not detect a difference in nitrite accumulation between the anti-miR-146a-5p and anti-miR-NC transfected cells, suggesting that the macrophages contributed the bulk of nitrites (Figures 4D,F).

**Figure 5:**
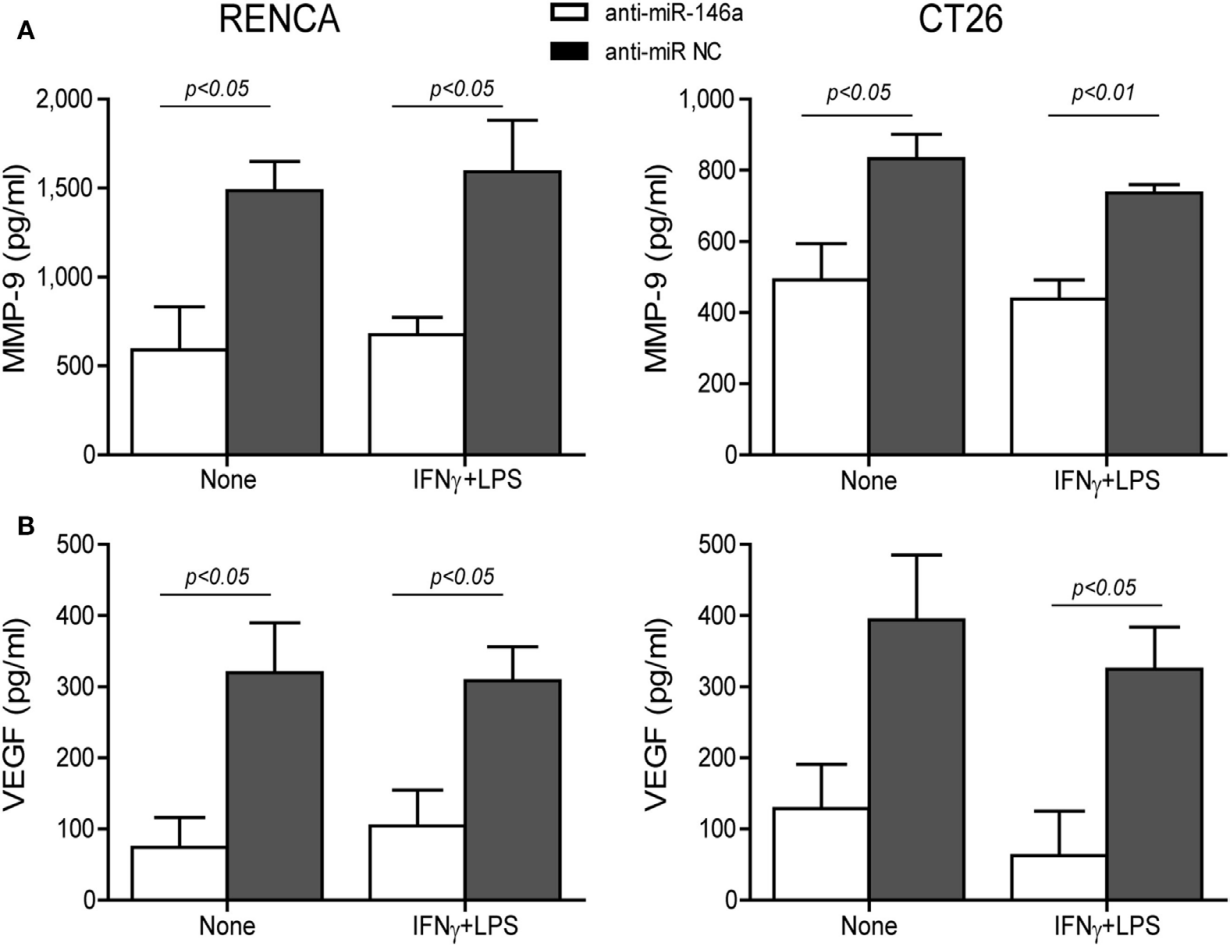
Neutralization of miR-146a-5p by its antagomir reduces matrix metalloproteinase-9 (MMP-9) and vascular endothelial growth factor (VEGF) concentrations in the supernatants. RENCA or CT26 cells (5 × 10^4^ cells) were transfected with anti-miR-146a-5p or with anti-miR-NC 24 h before they were stimulated with IFNγ (100 U/ml) and LPS (1 µg/ml). **(A)** Accumulation of MMP-9 and **(B)** VEGF in the supernatants was measured by ELISA (*n* = 4–5 in each group). The antagomir reduced MMP-9 and VEGF levels regardless of the combined stimulation.

**Figure 6:**
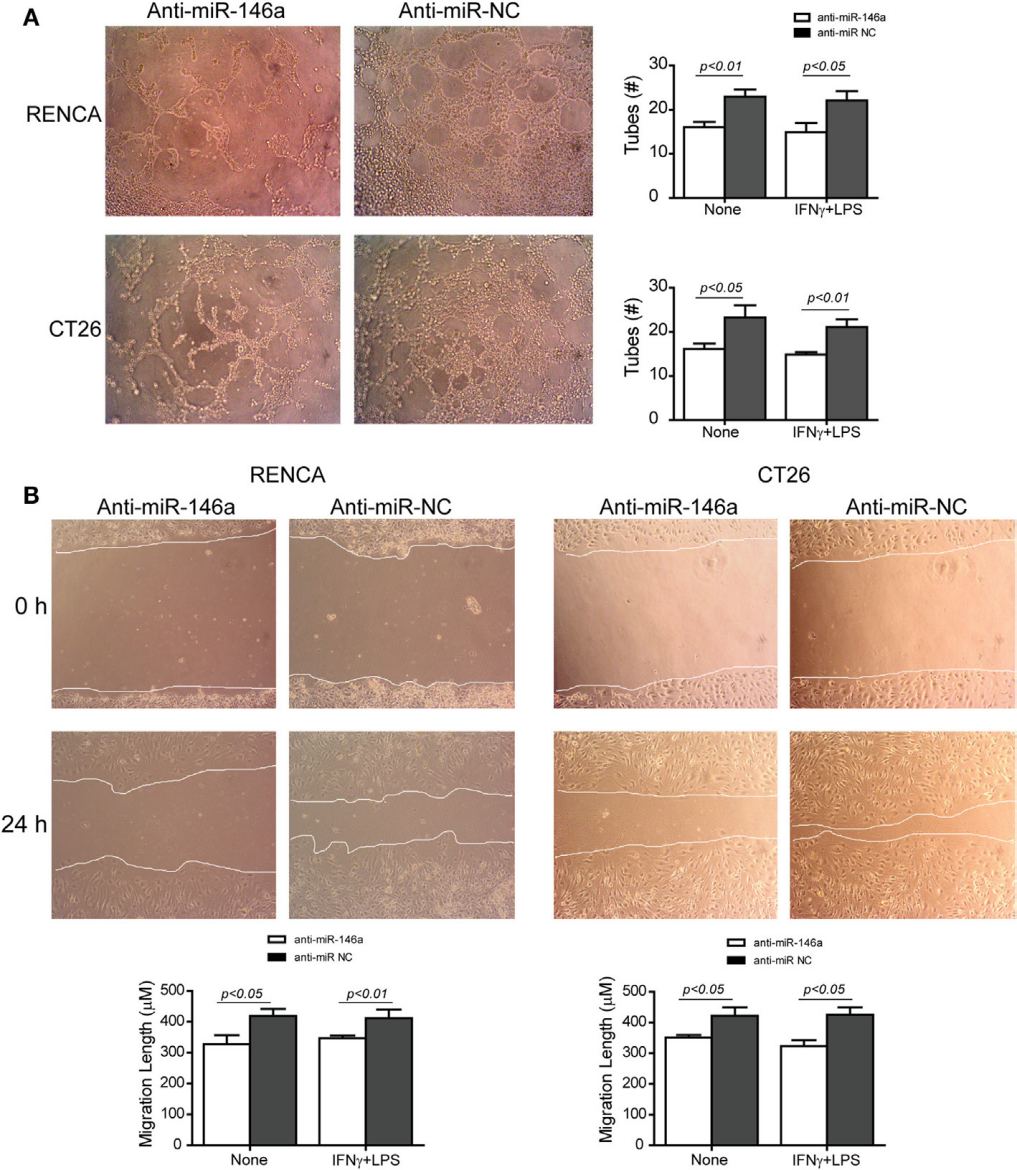
Neutralization of miR-146a-5p with its antagomir reduces angiogenesis. **(A)** Supernatants from single cultures of tumor cells obtained from the previous experiments (described in Figures 1–3) were diluted 1:2 in full medium and incubated with the mouse bEND3 endothelial cells (8 × 10^4^ cells) that were seeded on wells coated with Coultrex^®^. Images of the cells were taken after 6 h and the number of tubes with closed lumens was counted. **(B)** Confluent bEND3 endothelial cells were scratched and washed, and images were obtained at the beginning of the experiment (time 0 h) and 24 h later (magnification 20×). The length of endothelial cell migration was measured (*n* = 5–6 in each group).

Changes in the angiogenic activity of EMMPRIN were detected by the concentrations of its induced pro-angiogenic factors VEGF and MMP-9 in the supernatants of transfected cells. In comparison to cells transfected with the negative control, marked reduction in MMP-9 levels (about 2-folds, *p* < 0.05, Figure 5A) and VEGF levels (about 34-folds, *p* < 0.05, Figure 5B) were observed after transfection of anti-miR-146a-5p, regardless of the presence of the combined stimulation. Likewise, relative to cells transfected with the negative control, the supernatants from cells transfected with the antagomir caused a 40% reduction (*p* < 0.05) in the number of closed lumens (Figure 6A), and a 20–30% inhibition (*p* < 0.05) in endothelial cell proliferation and migration in the wound assay (Figure 6B). In both assays, the combined stimulation had no additional effects.

### *In Vivo* Neutralization of miR-146a-5p by Its Antagomir Reduces Tumor Growth and Angiogenesis and Increases Apoptosis

**Figure 7:**
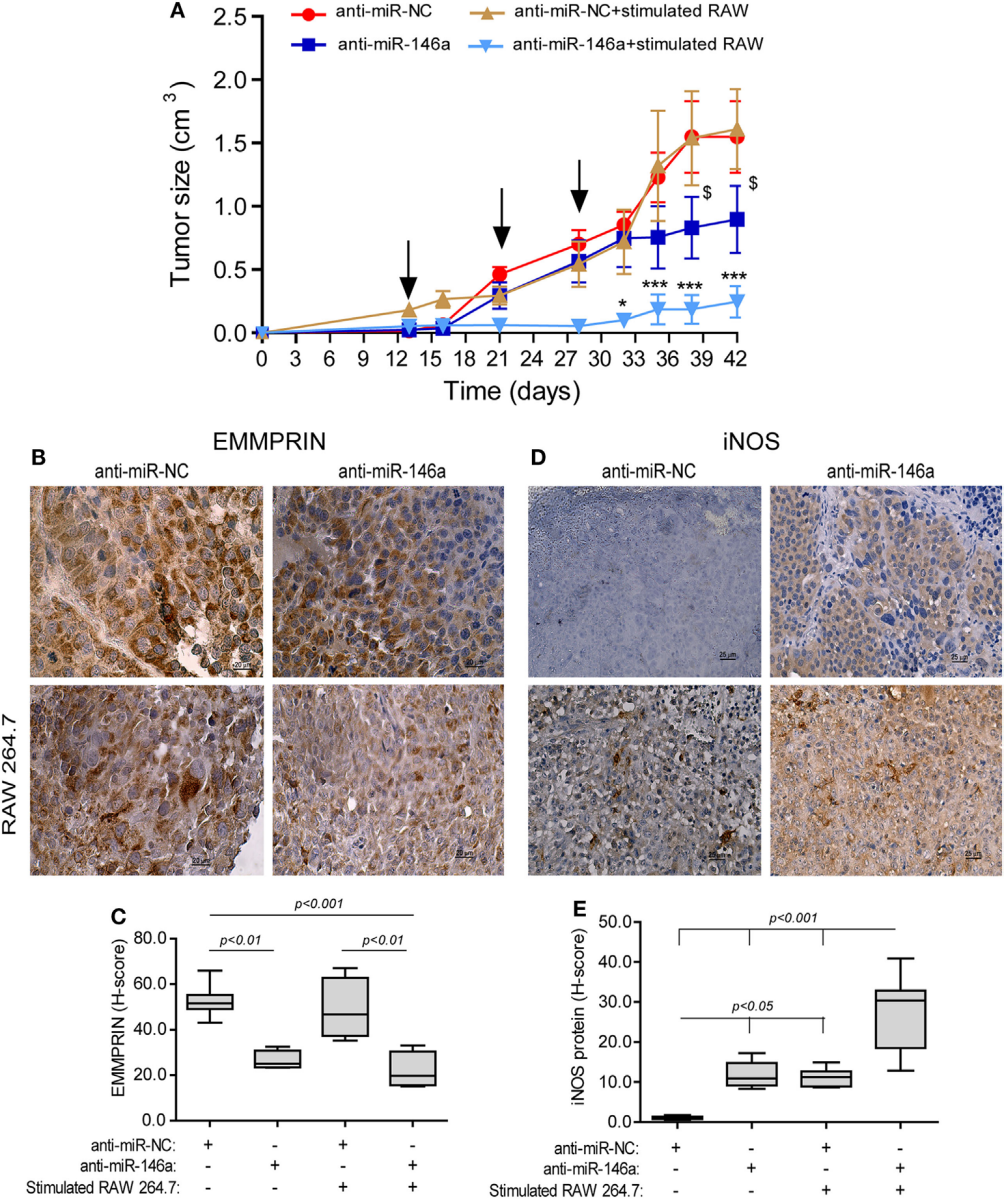
Neutralization of miR-146a-5p, together with pro-inflammatory stimulated macrophages, inhibits tumor growth. **(A)** RENCA cells (2 × 10^6^ cells) were injected to the flank of BALB/c mice. After tumors became palpable, mice were i.v. injected every 7 days (black arrows), with either anti-miR-146a-5p or its negative control anti-miR-NC (0.025 mg/g BW each), alone or together with injections of RAW 264.7 (10^6^ cells) that were stimulated *in vitro* with IFNγ (100 U/ml) and LPS (1 µg/ml) for 24 h prior to injection. *p < 0.05, ***p < 0.001 relative to the control group, $, *p* < 0.05 relative to the anti-miR-146a-5p group. Representative images of tissue sections immunohistochemically stained for **(B)** EMMPRIN protein expression and **(C)** its evaluation by the h-score, and **(D)** inducible nitric oxide synthase (iNOS) protein expression and **(E)** its evaluation by the *H*-score (*n* = 6 in the miR-NC+stimulated RAW 264.7 group, and *n* = 5 in each of the other groups, in two biological replicates).

To examine if the miR-146a-5p can be *in vivo* manipulated to reduce tumor size, we next subcutaneously implanted RENCA tumor cells in the syngeneic wild-type BALB/c mice. When tumors became palpable, we injected either the antagomir or its negative control to their circulation, with or without the simultaneous injection of RAW 264.7 cells that were previously *in vitro* stimulated with IFNγ and LPS for 24 h, to the rims of the tumors, where they would be least exposed to the hypoxic microenvironment. This was repeated three times every 7 days. Injection of the antagomir’s negative control (anti-miR-NC) with or without stimulated RAW264.7 cells did not affect tumor growth rate, and at the end of the experiment, the average tumor size was 1.54 ± 0.3 cm^3^ (Figure 7A). Injection of the antagomir alone resulted in a 1.4-folds reduction of tumor size (*p* < 0.05), whereas the combination of the antagomir and the stimulated macrophages resulted in a considerable slowing of the growth rate and about 6-folds reduction in tumor size (*p* < 0.001).

In mice injected with anti-miR-NC negative control, iNOS expression was not detected in the tumor cells, but macrophages that infiltrated the tumor after being injected to its rims expressed it in high levels, as evident by the intense staining (Figure 7D, low left panel). By contrast, iNOS expression was induced in RENCA tumor cells after anti-miR-146a-5p was injected i.v. (10-folds induction, *p* < 0.05, Figure 7D, right panels, Figure 7E).

EMMPRIN expression exhibited an inverse pattern to iNOS expression. Constitutive high expression levels of EMMPRIN were observed in the negative control group, and these were markedly reduced when anti-miR-146a-5p was injected, regardless of the injection of stimulated RAW 264.7 cells (about twofolds, *p* < 0.01, Figures 7B,C).

**Figure 8:**
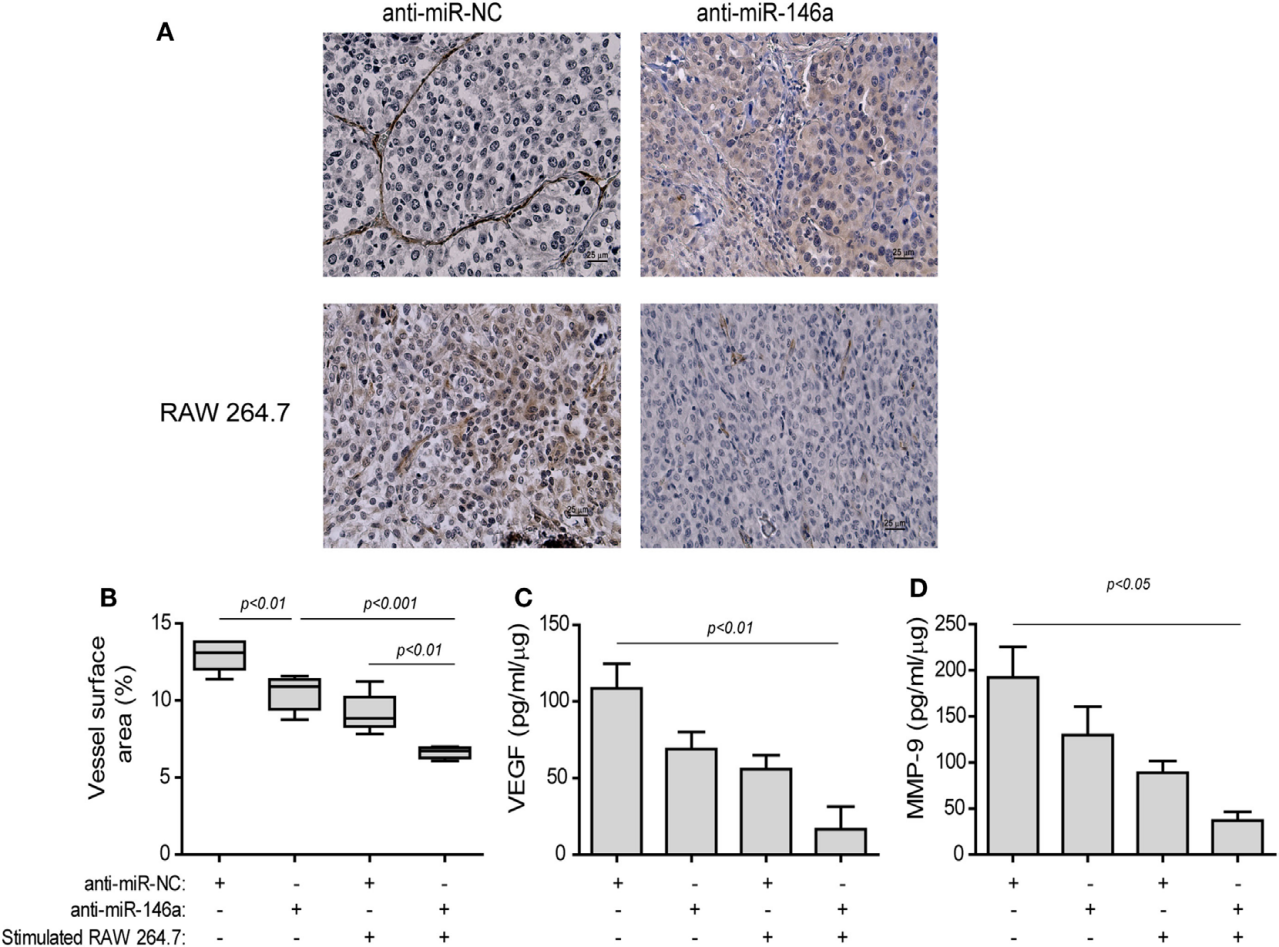
Stimulated macrophages and miR-146a-5p neutralization reduce angiogenesis. Tumors were harvested after 42 days, paraffin-embedded, sectioned, and stained for the expression of the endothelial cell marker CD31. **(A)** Representative images (scale bar = 25 μm) and **(B)** the estimation of vessel surface area. Tumor sections were lysed and concentrations of **(C)** matrix metalloproteinase-9 (MMP-9) and **(D)** vascular endothelial growth factor (VEGF) were determined by ELISA (*n* = 3–5 in each group).

The effects of the treatment on angiogenesis were first estimated by the change in the mean vessel density (MVD) by staining for the endothelial marker CD31 (Figure 8A). Blood vessels in the negative control group injected with the anti-miR-NC were long, branched, and continuous (Figure 8A, top left panel), whereas in the group injected with both the anti-miR-146a-5p and stimulated macrophages, blood vessels were short, discontinuous, and with wider gaps between them (Figure 8A, bottom right panel). The vessel surface area, a measure of MVD, was gradually reduced (Figure 8B), culminating in a twofold decrease relative to the group receiving both anti-miR-146a-5p and stimulated macrophages (*p* < 0.001). A reduction in the levels of the pro-angiogenic factors VEGF (by 6.7-folds, *p* < 0.01, Figure 8C) and MMP-9 (by 5-folds, *p* < 0.05, Figure 8D) was observed in the tumor lysates between the groups receiving the anti-miR-NC and the group receiving anti-miR-146a-5p and stimulated macrophages.

**Figure 9:**
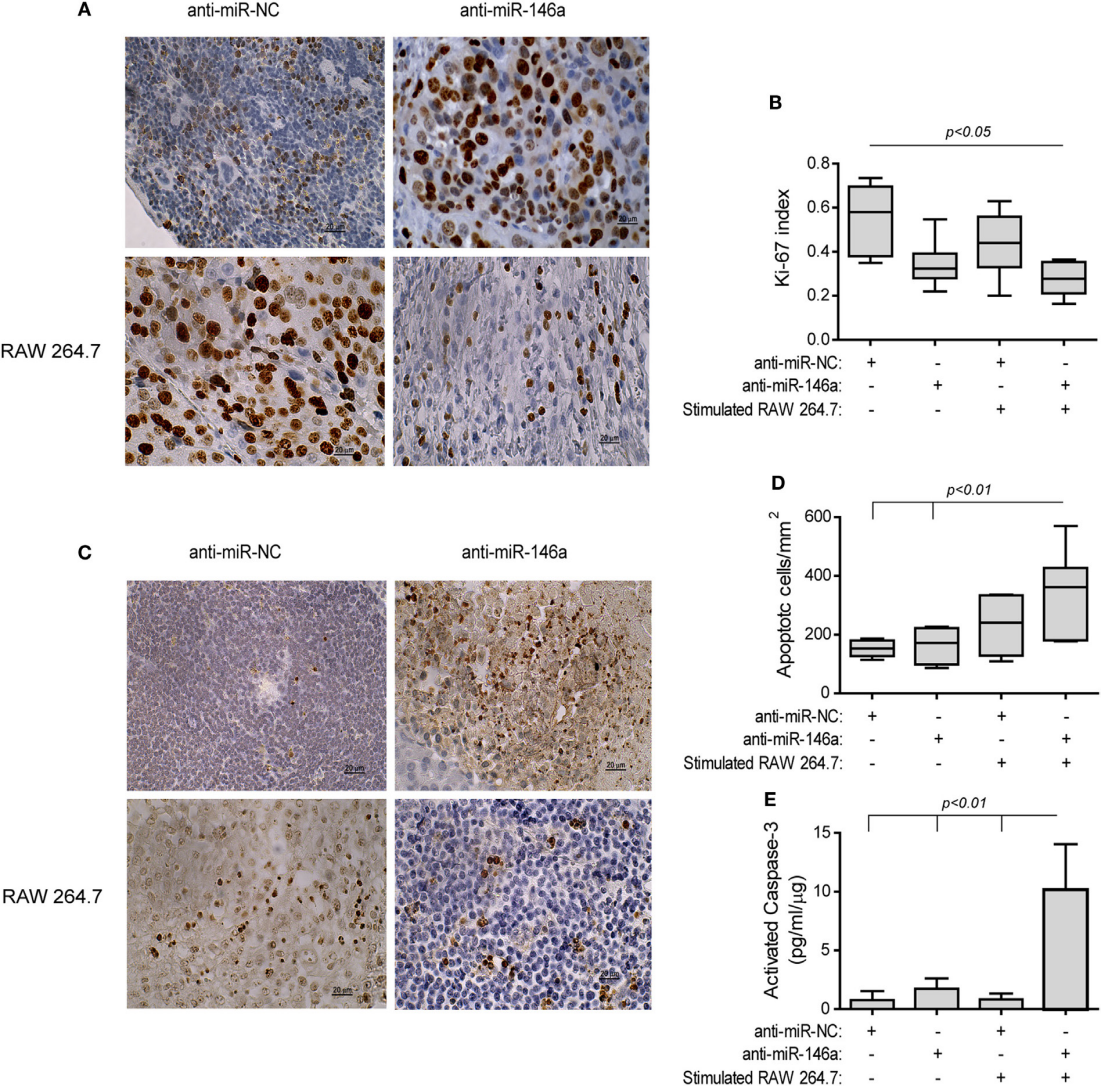
Stimulated macrophages and miR-146a-5p neutralization increases tumor cell apoptosis. Tumors were harvested, paraffin-embedded, sectioned, and stained for Ki-67 or DNA strand breaks (TUNEL assay). **(A)** Representative images of Ki-67 staining (scale bar = 20 μm) and **(B)** their quantitation. **(C)** Representative images of TUNEL assay and **(D)** their quantitation. **(E)** Tumor sections were lysed and concentrations of cleaved, activated caspase-3 were determined (*n* = 3–5 in each group).

The treatment with the antagomir and the stimulated macrophages reduced tumor cell proliferation (by twofolds, *p* < 0.05, Figures 9A,B) relative to the group receiving the anti-miR-NC alone, as assessed by the Ki-67 index. Complementarily, the rate of apoptosis was increased in this group, as evaluated by the TUNEL assay (2-folds, *p* < 0.01, Figures 9C,D) and the levels of activated caspase-3 (13-folds, *p* < 0.01, Figure 9E), relative to the negative control group.

**Figure 10:**
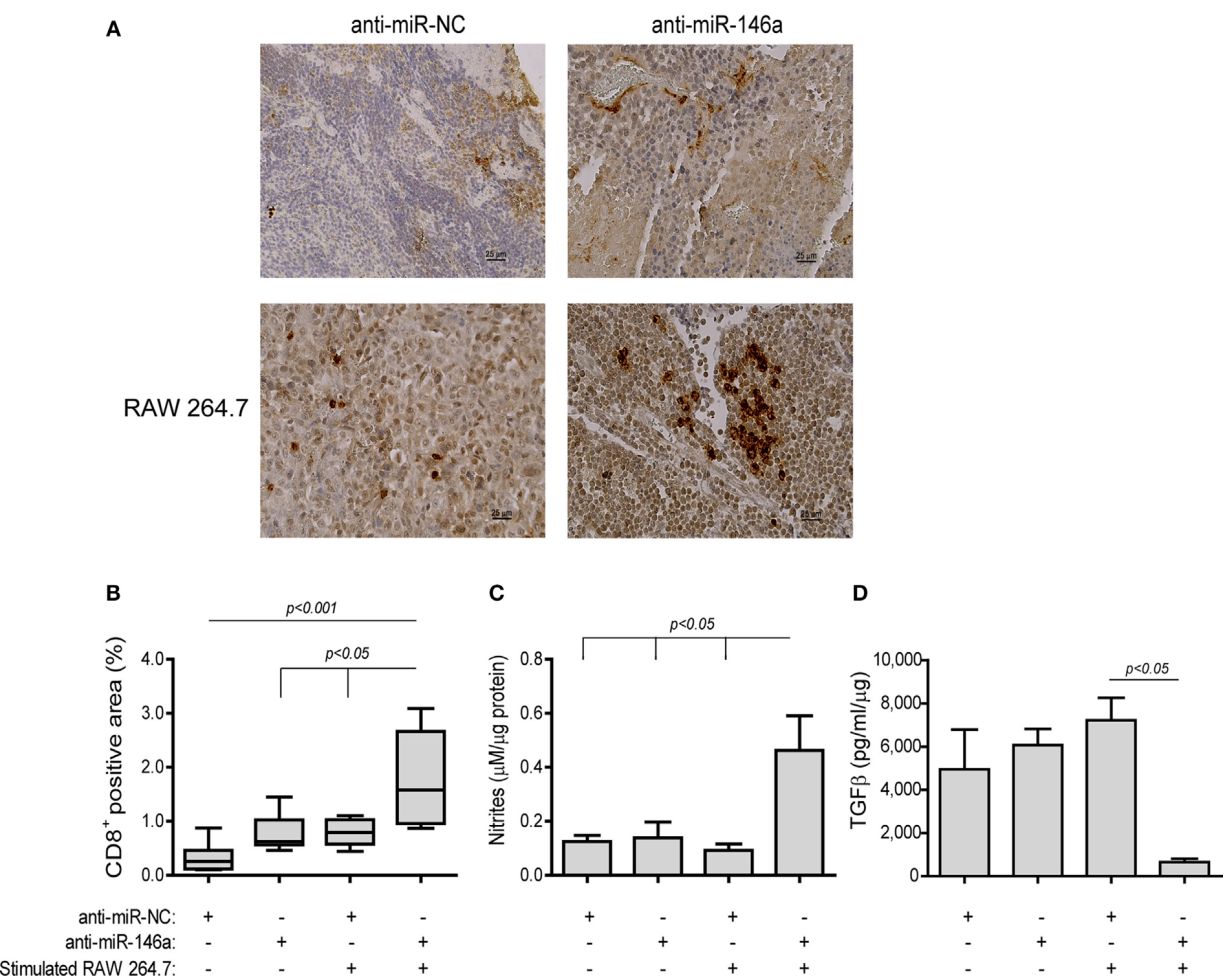
Stimulated macrophages and miR-146a-5p neutralization immune-modulate the tumor microenvironment. Tumors were harvested, paraffin-embedded, sectioned, and stained for the expression of CD8. **(A)** Representative images (scale bar = 25 μm) and **(B)** their quantitation. Tumor sections were lysed and concentrations of **(C)** nitrites and **(D)** TGFβ were determined (*n* = 3–5 in each group).

Lastly, to detect immune-related changes in the TME, we measured the infiltration of CD8^+^ T cells. In mice receiving anti-miR-NC alone CD8^+^ T cells were few and mostly limited to the rims of the tumors (Figure 10A, top left panel). By contrast, in the group receiving both the antagomir and the stimulated macrophages many CD8^+^ T cells infiltrated the tumor tissue, resulting in an increase in the positively stained area (by fivefolds, *p* < 0.001, Figure 10A, bottom right panel, Figure 10B). Since we injected stimulated macrophages into the rims of the tumor, we saw no point in staining for their presence. However, nitrite concentrations, reflecting the macrophage mode of activation, were measured in the tumor lysates and showed an increase (3.7-folds, *p* < 0.05, Figure 10C) in the group receiving both the antagomir and stimulated macrophages, although the absolute levels were low. The same group also showed reduced levels of TGFβ, a dominant M2-related cytokine, relative to the other groups [by 5.7-folds (Figure 10D), *p* < 0.05]. Hence, we believe that these changes indicate immune modulation and the alleviation of immune suppression.

## Discussion

In this study, we show that miR-146a-5p simultaneously and oppositely regulates the tumor cell expression of two key mediators of the inflammatory response in cancer: iNOS, which can potentially mediate tumor cell death, and EMMPRIN, which can enhance survival and angiogenesis though induction of VEGF and MMP-9. Thus, miR-146a-5p works as a regulatory switch between death and survival of tumor cells.

Here, we expand our previous findings in the mouse renal cell carcinoma RENCA (11) to the mouse colon cell carcinoma CT26, and show that these two tumor cells can escape macrophage-induced cell death if their iNOS protein expression is completely lost. Reduced iNOS expression in tumor cells has been associated with their ability to resist immune killing (10). We show that this ability is achieved by the post-translational inhibition exerted by high levels of miR-146a-5p. We show that the presence of M1-activated macrophages that produce high levels of NO is necessary for tumor cell death, but if the tumor cell does not endogenously produce NO, even in minute amounts, it remains resistant to the cytotoxic effects of NO produced by the macrophages. Although NO is a gaseous molecule that can easily transverse membranes, there is a distinction between its exogenous high production by the macrophages and the limited endogenous production by the tumor cells, which is critical in the determination of tumor cell survival or death. However, the precise mechanism that distinguishes between NO produced endogenously and exogenously is still unclear and merits further investigation.

Of note, NO has been shown to sensitize refractory tumors to radio- and chemotherapy (27–29), but increase their resistance to photodynamic therapy (30). However, the actual biological effect depends greatly on the concentrations of NO, the measure of hypoxia in the local site, and the cell type producing it. Our results, demonstrating sensitization of the RENCA and CT26 cells to macrophage-induced cell death only after restoration of iNOS and NO production, suggest that endogenous tumor NO production may activate pro-apoptotic pathways. Thus, in tumors that lost their iNOS and NO production, antagomir therapy may restore this production and serve to sensitize tumors to other treatment modalities, such as radio- or chemotherapies.

Simultaneously, high levels of miR-146a-5p also raise EMMPRIN expression in the same tumor cell, thus inducing angiogenesis by enhancing VEGF and MMP-9 secretion and by directly affecting endothelial cells, as observed in the *in vitro* tube formation and wound assays and suggested before (31). However, since we did not observe any presence of miR-146a-5p in the supernatants (data not shown), we negate the possibility that tumor cells export miR-146a-5p as a means to reprogram the neighboring macrophage. Thus, the effects of miR-146a-5p are limited to the tumor cells.

As miR-146a-5p emerged as a regulatory switch of tumor cell behavior, we next examined the potential therapeutic effects of neutralizing it by using a miR-146a-5p antagomir as a means to modulate tumor behavior and its microenvironment. First, we chose to inject anti-miR-146a-5p directly to the tail vein, as the chemical modification introduced to the mirVana antagomir increases its stability, and the tumor leaky vasculature enables its diffusion to the tumor cells [reviewed in Ref. (32–35)]. Next, we injected the stimulated macrophages into the rims of the tumor, as we have done before (11), to allow them to gradually exert their cytotoxic function, from the rims toward the tumor core, before they encounter the immunosuppressive effects of the hypoxic microenvironment.

Previously, the use of anti-miRNAs for therapy was hampered by several problems, especially the degradation of the anti-miRNA molecules in the circulation and their poor delivery to target sites. However, introduction of chemical modifications, such as the LNA technique that bridges the 2′-oxygen and the 4′-carbon, and the addition of a 2′-*O*-methyl group, markedly stabilized these molecules (22). Furthermore, anti-miRNAs were conjugated to different nanoparticles to improve delivery, including neutral lipid emulsions (e.g., DOPC), polyethylenimine, polyethylene glycol, and bacterium-derived particles coated with antibodies for specific target sites, to name just a few (22). Preclinical experiments using several specific modified anti-miRNAs delivered with different nanoparticles have already shown reduction in tumor growth, reduced metastasis, cell viability, and angiogenesis, without accumulating damage to normal tissues, indicating low toxicity (22, 23). However, antagomir therapy can be successfully used even without such delivery methods, and we show here that directly injecting the modified antagomir intravenously still inhibited tumor growth, bypassing this question.

Side effects or adverse responses were not reported when the expression of miR-146a was targeted in mice for therapy of different conditions, whether administered locally or systemically (36–38). However, in one case miR-146a antagomir successfully ameliorated the clinical symptoms in a myasthenia gravis model, but caused functional defects in B cells, including reduced antibody production, reduced number of plasma and memory cells, and reduced class switching (39). However, we do not believe that such effects, which are at the core of the B-cell driven autoimmune disease as myasthenia gravis, are relevant in our model, which rely mostly on the interaction between macrophages and tumor cells.

Since miR-146a is an inflammatory miRNA that regulates the NF-κB pathway among other influences, targeting it may be highly context dependent. In the *in vitro* experiments, we transfected only the tumor cells with the antagomir, causing a reduction of EMMPRIN expression and an increase in iNOS expression. However, when delivered systemically *in vivo*, both the tumor cells and the macrophages were exposed to the antagomir, and could potentially respond differently. In macrophages, the effects of the antagomir could potentially disrupt the negative regulation on the components of the NF-κB pathway TRAF6 and IRAK-1, which are verified targets of miR-146a-5p (40). Thus, the NF-κB pathway, which is needed for the induction of iNOS, should be enhanced, and the overall effects of iNOS expression should only increase. However, we could not conclusively discern whether the macrophages were in fact affected by the antagomir: first, because the levels of EMMPRIN expression were reduced in the presence of the antagomir to a level comparable to that of the negative control. Second, because the adoptively transferred macrophages were stimulated *ex vivo* with LPS, so their increased iNOS expression could be the result of either the combined stimulation or the effect of the antagomir.

We show that the combined treatment with anti-miR-146a-5p and the stimulated macrophages resulted in reduction of the anti-inflammatory cytokine TGFβ and concurrent increase in the infiltration of CD8^+^ cytotoxic T cells into the tumors. In addition, nitrites were accumulated in tumors receiving the combined stimulation, suggesting a shift in macrophage activation. We have recently shown that TGFβ is the dominant cytokine in the RENCA TME (41, 42). Therefore, its reduced levels together with the increased macrophage production of NO, altered the TME, alleviated immune suppression and allowed CD8^+^ T cells to infiltrate deep into the tumor and eradicate tumor cells. Signaling pathways leading to TGFβ activation are not yet fully understood, and although Smad4, which is part of the downstream TGFβ signaling pathway, has been identified as a direct target of miR-146a (43, 44), no regulatory loop has been established. Therefore, we could only speculate that either miR-146a indirectly affects TGFβ activation, or that the *ex vivo* stimulation of the macrophages that shifts them toward M1-activation, together with the administration of the antagomir, contributes, and gradually amplifies the reduction in TGFβ levels. Furthermore, we have not yet performed this experiment with implanted CT26 tumor cells, and due to their immunological status resulting from high expression of gp70, the product of the envelope protein of the murine leukemia virus retrovirus (45, 46), we cannot predict the outcome of such an experiment.

The concept of macrophage therapy was studied mostly in the 80s and 90s [reviewed in Ref. (47)]. The ability of macrophages to produce strong cytotoxic mediators, their ability to home directly into the core of tumors, and the easy protocols for their isolation from peripheral blood made them preferable instruments of therapy. However, all attempts to stimulate monocytes *ex vivo* with IFNγ or a combination of IFNγ and LPS, and then reinfuse them into the patient, failed. They did not produce beneficial effects in human patients, whereas in mice they exhibited a limited success to delay, but not regress, tumor growth (10). These disappointing results led researchers to abandon the concept. However, improved understanding of how the tumor-cell-driven immunosuppressive microenvironment shifts pro-inflammatory macrophages into a pro-angiogenic, M2-like mode of activation (6) may now enable us to alleviate immune suppression and allow macrophages and other immune cells to kill tumor cells, re-enabling this modified approach.

Our study demonstrates that inhibition of miR-146a-5p in combination with the adoptive transfer of stimulated macrophages can “turn off” angiogenesis and “turn on” tumor killing mechanisms such as iNOS, enabling the recruitment of additional activated immune cells that can now kill tumor cells. In other words, we can now re-visit macrophage therapy and improve it by manipulating miR-146a-5p levels. Naturally, such an approach should be further studied in different tumor models and eventually in clinical trials.

## Materials and Methods

### Cells

The tumorigenic mouse renal (RENCA, ATCC CRL-2947) and colon (CT26, ATCC CRL-2638) carcinoma cell lines were cultured in RPMI-1640 medium, 10% fetal calf serum (FCS), 1% l-Glutamine and antibiotics, with addition of 100 mM HEPES buffer (pH 7.4) for the RENCA cells, or 1% sodium pyruvate for the CT26 cells. The mouse TRAMP-C2 prostate cancer cell line (ATCC CRL-2731), the macrophage-like RAW 264.7 cell line (ATCC TIB-71), and the endothelial bEND3 cells (ATCC CRL-2299) were cultured in Dulbecco’s modified Eagle’s medium (DMEM) with 10% FCS, 1% l-glutamine and antibiotics, with addition of 5 µg/ml insulin and 10^−8^ mol/l methyltrienolone (R1881), the dihydrotestosteron analog (NLP005, Perkin-Elmer) for the TRAMP-C2 cells. All cell lines were used at passages 3–15 and regularly tested for morphological changes and presence of mycoplasma, RAW 264.7 cells were identified as macrophages by their ability to phagocytose zymosan particles, and tumor cells were tested as cells of epithelial origin by their expression of cytokeratin 18.

When indicated, cells were stimulated with IFNγ (100 U/ml, 485-MI-100, R&D systems, Minneapolis, MN, USA) and LPS (1 µg/ml, L-6529, *Escherichia coli* 055:B5, Sigma, St. Louis, MO, USA). To avoid immune stimulation or possible masking of signals by exogenous stimuli, cells were serum-starved before their exposure to the experimental conditions or their injection to mice.

### Reverse Transfection and Inhibition of miR-146a-5p

Reverse transfection and inhibition of miR-146a-5p were performed exactly as before (11) for both RENCA and CT26 cells, only that the mirVana anti-miR-146a-5p inhibitor™ (4464084, Ambion, Austin, TX, USA) or its negative control (4464076, anti-miR-NC, Ambion), at 30 nmol/l each, were used instead of the first-generation inhibitors.

### Quantitative Real-time PCR (qPCR) Analyses

Quantitative real-time PCR analyses were performed as described before (11). Total RNA was extracted from 10^6^ RENCA or CT26 cells using the RNA extraction kit (17200, Norgen biotek, ON, Canada), and 500 ng of total RNA were transcribed to cDNA using the High Capacity cDNA Reverse Transcription kit (4368814, Applied Biosystems, Foster City, CA, USA). Expression of iNOS and EMMPRIN mRNAs and their reference gene PBGD, or miR-146a-5p and its reference gene U6 were determined by qPCR using TaqMan assay on demand kit with the StepOne system (Applied Biosystems) in triplicates according to the manufacturer’s instructions.

### Determination of Nitrites, Western Blots Analyses, and Cytotoxic Assays

Determination of nitrites, western blots analyses, and cytotoxic assays were performed as before (11). The optical density of the bands in western blots was quantified using ImageJ. For the cytotoxic assays, the iNOS inhibitor 1,400 W (25 µM, W4262, Sigma) was used.

### Flow Cytometry

EMMPRIN expression was evaluated as before (17), using 1 µg of the FITC-conjugated anti-mouse CD147 or with its isotype control (123705, BioLegend, San Diego, CA, USA).

### Immunofluorescence

RENCA or CT26 cells (6 × 10^4^ cells) were transfected on cover slips with anti-miR-146a-5p as described above and fixed with cold methanol for 5 min at room temperature. Cells were permeabilized with 0.25% Triton-X 100 for 10 min, and incubated with blocking buffer (2% donkey normal serum, 0.1% Triton-X 100 in PBS) for 30 min at room temperature. Cells were stained with primary antibodies (rat anti-mouse EMMPRIN, MAB772, R&D systems, or rabbit anti-mouse iNOS, ab15323, Abcam, Cambridge, UK) diluted 1:250 in blocking buffer overnight at 4°C. Then secondary antibodies (Alexa 488-conjugated donkey anti-rat IgG, ab150153, Abcam, or Alexa 546-conjugated donkey anti-rabbit IgG, A10040, Thermo Fisher, Rockford, IL, USA) were diluted 1:500 in blocking buffer in the dark for 1 h at room temperature. Coverslips were mounted on a slide with fluoromount G. Three washes with PBS were applied after each step. Images were acquired by upright fluorescent trinocular microscope (Olympus BX-60, Tokyo, Japan) using the MS60 camera and the MShot Image Analysis System V1 (MSHOT, Guangzhou Micro-shot Technology Co., Guangzhou, China).

### ELISA

The mouse MMP-9, VEGF, TGFβ, and activated caspase-3 concentrations were determined as before (42). EMMPRIN concentrations were measured using with an ELISA kits (ab215405, Abcam) at a dilution of 1:200, according to the manufacturer’s instructions.

### *In Vitro* Wound Scratch Assay

*In vitro* wound scratch assay was performed as described before (17), with the mouse bEND3 endothelial cell monolayers (10^5^ cells) seeded in 24-well dishes and incubated with experimental supernatants derived from RENCA or CT26 cells transfected with anti-miR-146a-5p or its negative control (diluted 1:2 with medium). Images of the field of injury were acquired at the beginning of the experiment and after 24 h. The average distances between the two sides of the wound were measured along the scratch (at least eight locations per field) in both time periods using the ImagePro plus 4.5 software (Media Cybernetics, Inc., Rockville, MD, USA), and the difference, which reflects the length to which the cells migrated, is presented.

### *In Vitro* Tube Formation Assay

Coultrex^®^ reduced growth factor basement membrane extract (40 μl/well, 3433, Trevigen, Gaithersburg, MD, USA) was used to coat 96-well plates at 4°C, and incubated at 37°C for 2 h to polymerize. bEND3 cells (8 × 10^4^) were seeded in triplicates in DMEM with 2% FCS and experimental supernatants diluted 1:2 with medium. After 6 h, the number of closed lumens per microscopic field, representing tube-like structures, was counted in two separate fields.

### Experimental Mouse Model

Experimental mouse model BALB/c mice (female, 8 weeks old, Envigo, Jerusalem, Israel) were kept with a 12 h light/dark cycle and access to food and water *ad libitum*. Tumors were generated as before (11), and when they became palpable at day 14, mice were randomly assigned to four groups that received the following treatments every 7 days: three i.v. injections of 0.025 mg/g body weight of (a) anti-miR-146a-5p or (b) anti-miR-negative control (anti-miR-NC) to the tail vein. Groups (c) and (d) were treated as groups (a) and (b), respectively, with the addition of 10^6^ RAW 264.7 cells stimulated with IFNγ (100 U/ml) and LPS (1 µg/ml) for 24 h, injected to the rims of the tumors. Tumors were measured every 3–4 days and their volume calculated (length × width × 0.5 cm^3^). At the end of the experiment, or when tumors were greater than 1.5 cm^3^, mice were euthanized and their tumor tissues were harvested. Part of the tumor was freshly frozen for evaluation of nitrite and cytokine concentrations in tumor lysates, while other parts were formalin-fixed and paraffin-embedded for later analysis by immunohistochemical staining.

### Immunohistochemistry and Immune Reactive Score

Immunohistochemistry and assigning an immune reactive score was performed as described in Ref. (42). Antigen retrieval for iNOS was performed by microwave heating in citrate buffer pH 6.0, and the antibody used was rabbit anti-iNOS (Abcam). All sections were viewed under the bright field trinocular microscope (Olympus BX-60, Tokyo, Japan) and images were acquired with the MS60 camera and the MShot Image Analysis System V1 (MSHOT, Guangzhou Micro-shot Technology Co., Guangzhou, China). Vessel densities assessed by CD31 staining and by using a Weibel grid to calculate vessel surface area (48), and the fraction of Ki-67-positive tumor cells was calculated by the digital image analysis web application ImageJS (49). EMMPRIN and iNOS expression were assessed using the modified H-score, which assigns an immune reactive score on a continuous scale of 0–300, based on the percentage of positive cells expressing the protein at different intensities. Staining was divided into three categories: 1 for “light staining,” 2 for “intermediate staining,” and 3 for “strong staining.” The percentage of positive cells was determined according to the positive surface area of cells measured with ImagePro plus 4.5 software, and the score was calculated using the formula: 1 × (%1 positive cells) + 2 × (%2 positive cells) + 3 × (%3 positive cells).

### Statistical Analyses

All values are presented as means ± SE. Significance between two groups was determined using the two-tailed unpaired *t*-test. Differences between three or more experimental groups were analyzed using one-way analysis of variance (ANOVA) and the *post hoc* Bonferroni’s multiple comparison tests, and the two-way ANOVA following Bonferroni’s post-tests for comparing time and groups. *P* values exceeding 0.05 were not considered significant.

## Ethics Statement

Mice were cared for in accordance with the procedures outlined in the NIH Guideline for the Care and Use of laboratory Animals, and all experiments were performed under the approved protocol (IL-121-12-11) issued by the Animal Care and Use Committee of the Technion-Israel Institute of Technology.

## Author Contributions

ES performed the experiments; VB was in charge of the immunohistochemical staining; MMR performed the *in vivo* experiments; and MAR designed the study, analyzed and interpreted the results, and wrote the manuscript.

## Conflict of Interest Statement

The authors declare that the research was conducted in the absence of any commercial or financial relationships that could be construed as a potential conflict of interest. The reviewer PB and handling editor declared their shared affiliation.
